# Scientific civility and academic performance

**DOI:** 10.1101/2023.01.26.525747

**Published:** 2023-01-27

**Authors:** Emma Camacho, Quigly Dragotakes, Isabella Hartshorn, Arturo Casadevall, Daniel L Buccino

**Affiliations:** 1 W. Harry Feinstone Department of Molecular Microbiology and Immunology, Bloomberg School of Public Health, Johns Hopkins University, Baltimore, Maryland 21205; 2 Department of Psychiatry and Behavioral Sciences, School of Medicine, Johns Hopkins University, Baltimore, Maryland 21205

**Keywords:** Civility, Collaboration, Productivity, Inequalities

## Abstract

In modern science, interdisciplinary and collaborative research is encouraged among scientists to solve complex problems. However, when the time comes to measure an individual’s academic productivity, collaborative efforts are hard to conceptualize and quantify. In this study, we hypothesized that a social behavior coined “scientific civility”, which encompasses civility, collaboration, cooperation, or a combination of these, enhances an individual’s productivity influencing their academic performance. To facilitate recognition of this unique attribute within the scientific environment, we developed a new indicator: the *C* score. We examined publicly available data from 579 academic scientists at the individual-level, focusing on their scholarly output and collaborative networks as a function of geographic distribution and time. Our findings demonstrate that the *C* score gauges academic performance from an integral perspective based on a synergistic interaction between productivity and collaborative networks, prevailing over institutionally limited economic resources and minimizing inequalities related to the length of individual’s academic career, field of investigation, and gender.

## Introduction

The American workplace (1) is among the most diverse in the world and the academic milieu is no different. Whether in a laboratory, a clinic, or a classroom, successful scientists in academia must be able to operate effectively in a diverse and dynamic environment, whether they be knowledge generators or disseminators, clinicians, educators, or administrators. Workplace civility has been defined as behaviors that help to preserve the norms of mutual respect and collaboration among co-workers (2, 3). Civility is more than being polite, it is the intentional choice of seeking common ground, disagreeing without disrespect, and genuinely caring about other people’s needs and beliefs (4). The core constituents of civility – relational competence and purposeful poise (5)– are essential for productivity and morale where there may be individuals with four to five decades of age difference working together, as well as people of different races, nationalities, gender identities, sexual orientations, and many other sometimes competing equity interests. To be successful in the academic workplace requires the capacity for collaboration, collegiality, openness, and respect for oneself and others, especially coworkers, co-investigators, and learners.

Traditionally, use of bibliometric indicators to assess scientific productivity and academic performance has revolved around quantitative measures regarding number of publications (6, 7), citation counts (8), and impact factor of the journal where research work was published (9). Scientific success has been historically associated with academic tenure, securing external funding, and recognition by prestigious awards (e.g., Nobel Laureate) (10) within a tremendously competitive environment. When the time comes to measure academic productivity, promotions committees tend to overvalue the monadic scholar “genius” and potentially undervalue what appear to be overly collaborative or group enterprises (11).

In recent years, the notion of “team science” has been defined as collaborative efforts (interdisciplinary or multidisciplinary) between and among groups of scientists who work together to solve a particular scientific challenge. Often, this approach tends to be limited in time (defined duration) and/or space (a building with large open labs) (12, 13). In this study we investigated the hypothesis that the academic performance and impact of an individual’s scientific output was strongly associated with a social behavior we called “scientific civility” to capture the constellation of attributes that contribute to successful human interactions in science. To quantify this observation, we developed an index termed the *C* score, where the C stands for *C*ivility, *C*ooperation, *C*ollaboration, or a combination of these, which for simplicity we shorthand to civility with the understanding that it encompasses the other C’s. We propose that the *C* score provides an integrative and holistic approach to assess an individual’s academic performance as the result of their successful scientific collaborations. This characteristic is an important attribute of scientific citizenship whose measure should be considered a valuable tool in professional and promotion decision-making scenarios.

## Materials and Methods

### Sample.

The analysis was conducted from 2006 to 2020 with a total of 579 academic individuals. The study relied on analysis of publicly available bibliometric data and no interactions of any kind (e.g., face-to-face, on paper, or in electronic realms) with any individual was conducted. Nonetheless, the study was submitted to The Johns Hopkins Medicine Institutional Review Boards (JHM IRBs) for reviewing and qualified as exempt research under the U.S. Department of Health and Human Services (DHHS) regulations.

### Design.

We developed a measurement— the *C* score. Using a binary scoring system, we operationalize the abstract construct of “scientific civility” into a measurable observation that ranges from 1 to 11 points. At a defined time of a scientist’s career, their *C* score is determined as *C* = *SOY* + *CN*, where SOY (Scholarly Output per Year) corresponded to academic productivity and CN (Collaborative Networks) corresponded to collaborative efforts over time and geographic distribution. SOY and CN are weighted variables that account for 45% and 55% of the total score, respectively.

**Figure 1.**
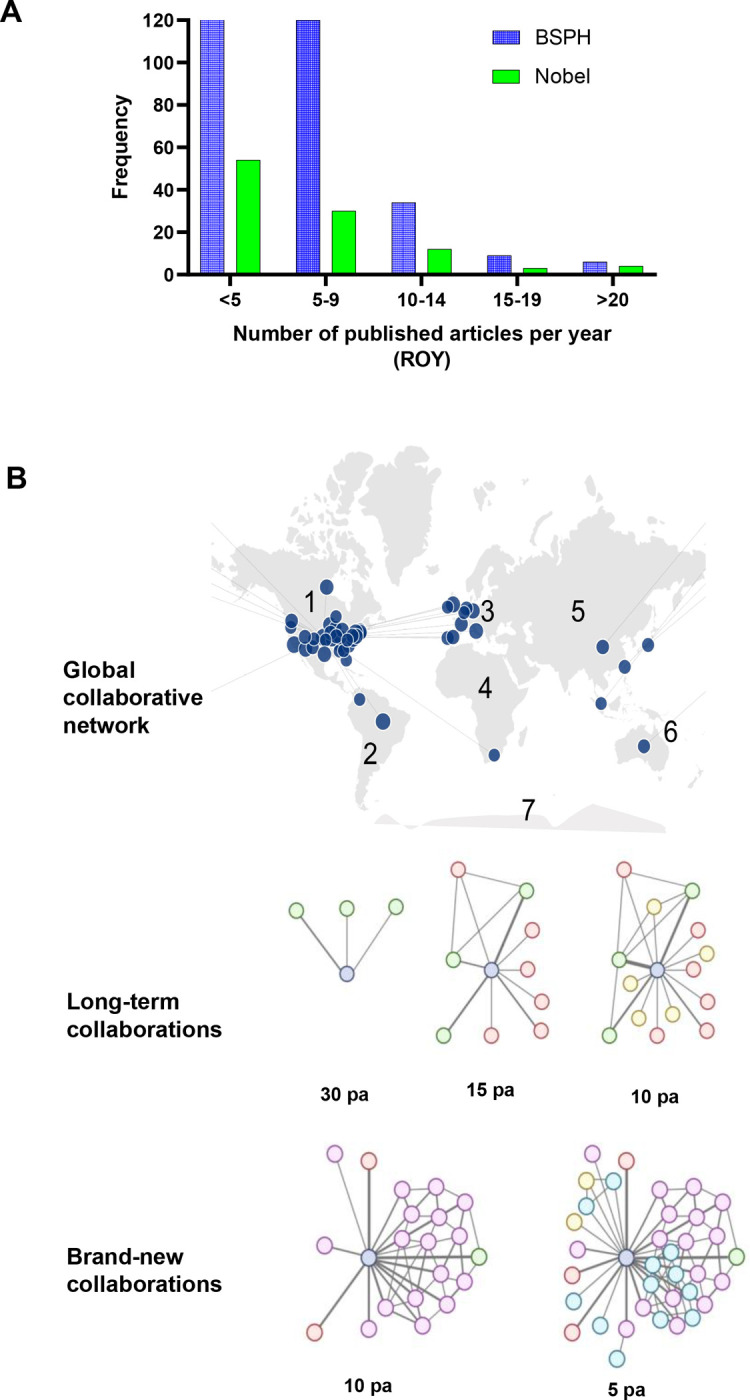
Scholarly output per year (SOY) and collaborative networks (CN) are independent and synergistic variables that contribute to a scientist academic performance. **A)** Distribution of research productivity based on the number of published articles per year (ROY) among Professors from the Johns Hopkins University Bloomberg School of Public Health (BSPH) and Nobel Laureates in Chemistry, Physics, Economic Sciences, and Physiology from 2010 to 2020. **B)** Diagram of collaborative efforts assessed as a function of geographic distribution and time. *Upper panel*, Global collaborative network represents the ability to establish collaborative projects with culturally diverse individuals based on institutions across the seven continents. *Middle panel*, Long-term collaborations captures the ability to develop and maintain a prolific productivity with unique scientists over a decade. *Bottom panel*, Brand-new collaborations refers to the ability to intellectually engage with new investigators. Thickness of interconnecting lines represents level of engagement between individuals in terms of number of published articles (pa). Light blue represents individual’s network that is being assessed.

In this study, the scholarly output corresponded to Research Output per Year (ROY) as the total number of publications generated during a scientist’s academic career normalized by year. To calculate an individual’s *C* score, for the ROY component of the equation a point is given according to the level of productivity that matches the ROY ≥20, ≥15, ≥10, ≥5, and >0. These levels were defined from previous assessment of the ROY distribution within two diverse and interesting sample populations (i.e., Tenure-Track Professors from The Johns Hopkins University Bloomberg School of Public Health and Nobel Laureates from 2010–2020 in four distinct categories) (Fig. 1A). In both samples, the number of individuals decreased as the level of productivity increased. Therefore, to reward the highest productivity level, a maximum of 5 points could be accounted for this variable. For the CN component, we created sub-categories that measured levels of engagement as a function of geographic distribution and time (Fig. 1B). These are the following:

1. Global collaboration network, accounts for the existence of collaborative projects with investigators affiliated with institutions from diverse cultural backgrounds using as reference the seven continents model: North America, South America, Europe, Asia, Africa, Oceania, and Antarctica. A point was given to an individual, if collaborative efforts with other scientists located in ≥4 continents have occurred during the length of their career.
2. Long-term collaborations sustained within the oldest 12 years or from the start of the academic career. For this study we used the time frame 2006–2017. This refers to the scientist’s ability to develop and maintain a prolific productivity with one or more investigators over a decade. Points were assigned according to the level of productivity with unique investigators: Minimum of 30 published articles (pa), Minimum of 15 pa, and Minimum of 10 pa. A maximum of 3 points could be accounted for this criterion.
3. Brand-new collaborations developed within the last 3 years (2018–2020). This refers to the scientist’s ability to intellectually engage with new investigators to develop, publish, and maintain collaborative projects that further expand their main research area and/or create a new line of investigation. As done previously, points were assigned according to the level of productivity with unique investigators: Minimum of 10 pa, and Minimum of 5 pa. A maximum of 2 points could be accounted for this criterion.

### Data.

To collect the information used for this study, we used the SCOPUS database curated by Elsevier. We performed SCOPUS author, document, and affiliate searches to retrieve the entire body of scholarly literature associated with each individual via unique Scopus ID. This data was collected through the SCOPUS APIs using the Elsapy Python package (version 0.5.0). Collaborator affiliations for calculating global network scores are based on the affiliation of each author at the time of publication for each given article. Data used for this study were downloaded between 2021–2022 and included the full years between 2006 and 2020. Data was available from The Center for Scientific Integrity, the parent nonprofit organization of Retraction Watch, subject to a standard data use agreement. Gender was assumed by searching for images of an individual’s name using the Google search engine. For one individual, we were not able to assign a gender. All the primary information used in this study was obtained from publicly available databases, such as university faculty directories, Nobel Prize winner lists, etc. Quality of the code was assessed with a sample population (around 15%) by accessing the SCOPUS database using an individual’s full name and manually measuring all parameters associated to the ROY and CN variables.

### Statistical analysis.

Relationships between the *C* score and *h* index were calculated using a Spearman correlation coefficient between the two values for each given dataset; 95% confidence intervals were used to summarized association. Correlations were not calculated for any grouping that included < 4 individuals. Analyses were performed in R version 4.1.3 (R Development Core Team, 2013).

## Results

**Figure 2.**
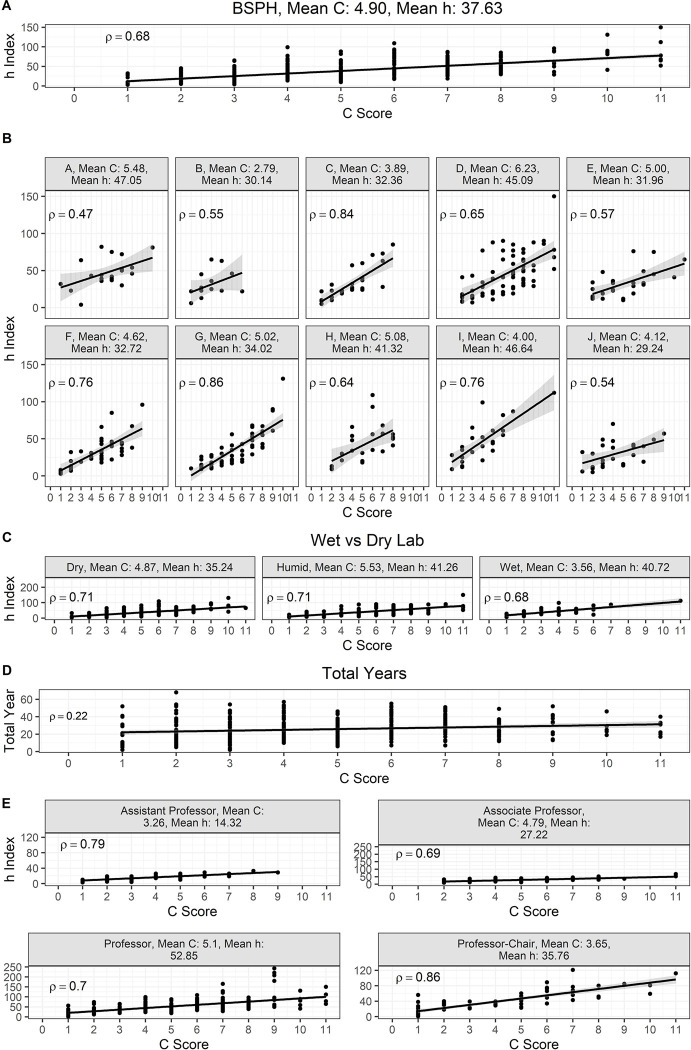
The *C* score is a useful index to assess academic performance. A scientist’s *h* index shows a positive strong relationship with the *C* score. **A)** Case study of The Johns Hopkins Bloomberg School of Public Health (BSPH). **B)** Case of 10 individual departments that comprise the BSPH. **C)** Profile by grouping departments according to the type of laboratory environment; **D)** The *C* score is not affected by the length of an individual’s academic career. **E)** The *C* score serves as potential tool to track advancement of an individual’s academic career.

We hypothesized that sustained collaborative networks are an important characteristic of successful academic/scientific performance. To validate or refute this hypothesis, first we set out to analyze the profiles of professors from our own institution, the Johns Hopkins University (JHU). These included all tenure-track professors (i.e., Assistant Professor, Associate Professor, and Professor) with a primary affiliation to the Bloomberg School of Public Health (BSPH), the first and largest school of public health in the United States comprised of 10 academic departments, which were de-identified and listed from A to J. A Spearman correlation (ρ) was computed to investigate whether there is a relationship between the *C* score and *h* index (14), a citation-based standard metric for quantifying the research output and impact of a scientist’s published work. The analysis revealed a strong, positive correlation between the two variables, which was statistically highly significant (ρ = 0.68, *N* = 323, *P* < 0.0001). Hence, the *h* index was associated with the *C* score. Mean *C* score and mean *h* index were 4.9 and 37.63, respectively (Fig.2A). Given the diverse nature of research areas and approaches to study problems within the BSPH, we assessed the *h* index and *C* score relationship by individual departments. We found that there was a strong to very strong correlation between these two variables, lowest ρ = 0.47, *P* = 0.0508 to highest ρ = 0.86, *P* < 0.0001 for A and G departments, respectively (Fig. 2B). Based on the type of lab environment, we grouped these departments intro dry, humid, or wet labs. We considered as dry labs those in the departments of: Biostatistics (BIO), Health, Behavior and Society (HBS), Health Policy and Management (HPM), International Health (IH), Mental Health (MH), and Population, Family and Reproductive Health (PFRH); as humid labs those in departments of: Environmental Health and Engineering (EHE) and Epidemiology (EPI); and as wet labs those in departments of: Molecular Microbiology and Immunology (MMI) and Biochemistry and Molecular Biology (BMB). Further distinction between the type of lab environment (dry, humid, or wet) also demonstrated a strong to very strong positive correlation between *h* index and *C* score, ρ = 0.71, *N* = 191, *P* < 0.0001; ρ = 0.71, *N* = 93, *P* < 0.0001; ρ = 0.68, *N* = 39, *P* < 0.0001, respectively (Fig. 2C). A non-parametric analysis using a Kruskal-Wallis test demonstrated that differences in the mean *C* score between groups were significant (Dry vs. Wet, *P* = 0.0027, Humid vs. Wet, *P* < 0.0001), except for Dry vs. Humid. Next, we tested whether the number of years in academia since a scientist published their first article would impact the *C* score. For this analysis, we also included other JHU professors not affiliated to the BSPH including the School of Medicine, Whiting School of Engineering, and Krieger School of Arts and Sciences. The length of the academic career showed a weak association with the *C* score, suggesting that a scientist’s academic performance is mostly driven by other factors (ρ = 0.21 (none), *N* = 406, *P* < 0.0001 (Fig. 2D). Interestingly, we noted that ranks within the academic tenure-track of Assistant Professor (Assist. Prof.), Associate Professor (Assoc. Prof.), Professor (Prof.), and Professor-Chair (Prof.-Chair) each revealed a strong to very strong, positive correlation with the *C* score that was highly significant (ρ = 0.79, *N* = 76, *P* < 0.0001; ρ = 0.69, *N* = 82, *P* < 0.0001; ρ = 0.7, *N* = 248, *P* < 0.0001, ρ = 0.86, *N* = 66, *P* < 0.0001, and respectively) (Fig. 2E). Significant differences on the mean *C* score were found between the ranks (Assist. Prof. vs Assoc. Prof., *P* = 0.0009; Assist. Prof. vs Prof., *P* < 0.0001; and Assoc. Prof. vs Prof. Chair, *P* < 0.0001, Kruskal-Wallis test) (*SI Appendix*, Fig. S1). In addition, mean *C* score-*h* index pairings were 3.26–14.32, 4.79–27.22, and 5.1–52.85 for Assist. Prof., Assoc. Prof., and Prof., respectively, suggesting the *C* score’s potential value for assessing career advancement.

**Figure 3.**
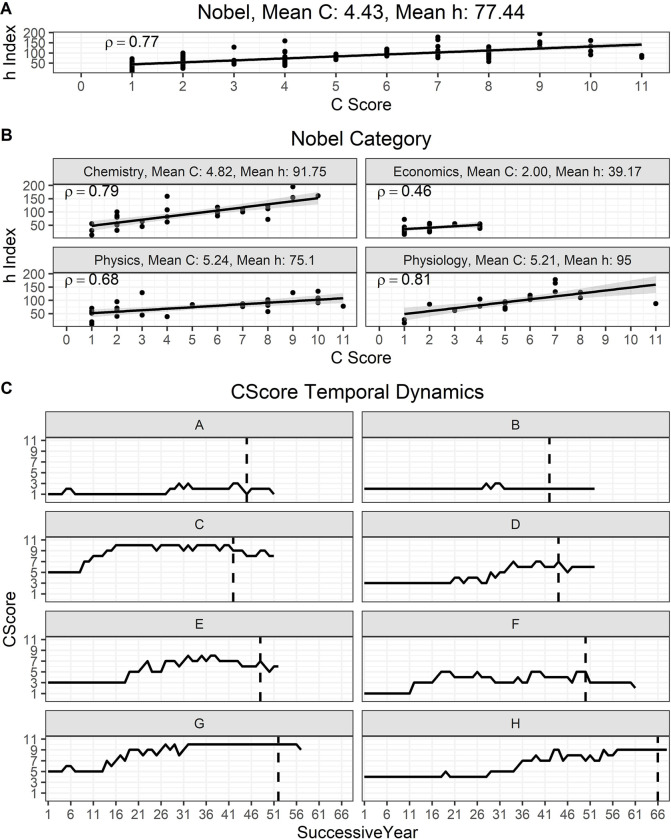
The *C* score reflects the likelihood of being recognized by a prestigious award and does not reflect a cumulative pattern. **A)** Case study of Nobel Laurates from 2010–2020 in four categories. **B)** Individual categories of Nobel Prizes also demonstrated a strong to very strong association between the two variables. **C)** The *C* score dynamics during the length of a scientist’s career reflects distinct levels of engagement with collaborative projects.

We also investigated whether a high *C* score was associated with individuals whose significant scientific achievements were recognized by a Nobel Prize. For this purpose, we analyzed the profiles from 103 Nobel Laureates from 2010–2020 in the categories of Physiology, Chemistry, Physics, and Economic Sciences. Like the JHU professors, for Nobel Laureates the *h* index and *C* score were positively correlated, ρ = 0.77, *P* < 0.0001 (Fig. 3A). Mean *C* score-*h* index pairings for this group were 4.43–77.44 while by individual categories were 4.82–91.75, 5.24 –75.1, 2.00–39.17, and 5.21–95 for Chemistry, Physics, Economic Sciences, and Physiology, respectively (Fig. 3B). A strong to very strong positive relationship between the two variables was found for all four categories, including those in the field of economics (ρ = 0.46, *N* = 22, *P* = 0.0145). For both individual and shared Nobel Prizes, we found that the *C* score of awardees spanned the whole range of the index (from 1 to 11). This implies that Nobel recognition can occur at the productivity peak of an individual, long after peak productivity, or even when the scientist has only published a few articles and their discoveries were considered to confer the greatest benefit on mankind. Shared Nobel Prizes reflect this pattern (*SI Appendix*, Table S1). Interestingly, we noted that along a scientist’s career the *C* score could exhibit a dynamic behavior. To illustrate this observation, we selected 8 Nobel Laureates from the same category whose career length was at least 50 years (Fig. 3C). Over the 50-year period of these individual’s scientific careers, the *C* score increased, decreased, or remained steady. These distinct patterns reflect diverse levels of engagement in collaborative projects.

**Figure 4.**
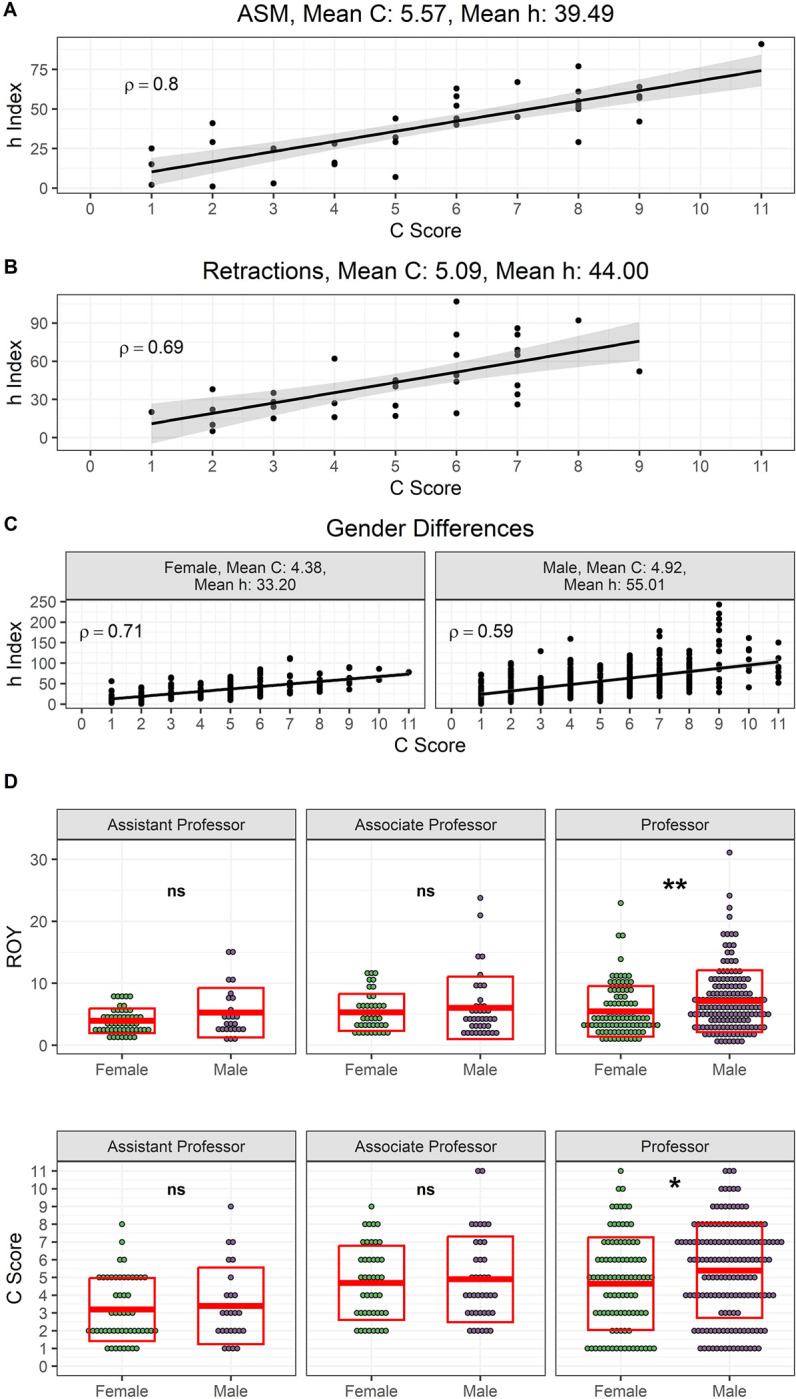
Active collaborative networks can bolster an individual’s academic performance. **A)** Case study of ASM International Fellows from Middle Income Economies countries. **B)** Case study of retracted authors. **C)** Academic performance in terms of the *C* score demonstrates no gender inequalities. **D)** Collaborative efforts in younger generations of academic professors across national and international borders can neutralize gender disparities in research productivity.

Furthermore, since the productivity and relevance of a scientist’s research output is highly dependent on the economic resources of the institution with which he or she is affiliated (15), we assessed whether the impact and record of scientific achievements among researchers from countries with limited economic resources also associates with high *C* scores. Starting with the premise that the *C* score was useful to estimate the broad impact of a scientist’s published work from the biological sciences, we selected a group of individuals from outside the USA who were elected to the American Academy of Microbiology (16). The Academy is the honorific leadership group within the American Society for Microbiology (ASM), one of the largest life science societies in the world (17). To be elected for fellowship, two basic qualifications must be met: 1) Recognition at the national or international level, and 2) Outstanding and original contributions to the microbial sciences. In addition, we considered culturally diverse individuals from nine countries defined as Middle-Income Countries (MICs) according to the World Bank (e.g., Argentina, Brazil, South Africa, India, Mexico, Lebanon, Russia, China, and Bangladesh). These countries are home to 75% of the world’s population and 62% of the world’s poor (18). Like the groups previously analyzed, we found a strong positive correlation between the *C* score and *h* index, ρ = 0.8, *N* = 37, *P* < 0.0001. Mean *C* score and *h* index were 5.57 and 39.49, respectively (Fig. 4A). On another measure, we examined the *C* score from authors involved in retracted articles. Using the retraction watch database (19), we selected individuals who were affiliated with institutions in the USA and co-authored research articles retracted between 01/01/2015 to 12/31/2022 due to falsification/fabrication of image or paper mill. These scientists revealed a strong association between *h* index and *C* score, ρ = 0.69, *N* = 35, *P* < 0.0001. Mean *C* score and *h* index were 5.09 and 44, respectively (Fig. 4B). In addition, we tested whether the association between *h* index and *C* score was influenced by gender. All individuals, except one for whom we couldn’t assign a gender, were included in the analysis. Cumulative impact and relevance of the research output from both female-assigned and male-assigned scientists positively correlated with their *C* score, ρ = 0.71, *N* = 212, *P* < 0.0001 and ρ = 0.59, *N* = 365, *P* < 0.0001, respectively (Fig. 4C). While the differences on the mean *h* index for female and male were highly significant 33.2 vs 55.01, respectively, *P* < 0.0001, Mann-Whitney test) (*SI Appendix,* Fig. S2A), the mean *C* score demonstrated a subtle significant difference 4.38 vs 4.92 for female and male, respectively, *P* = 0.03, Mann-Whitney test) (*SI Appendix,* Fig. S2B). Overall, male scientists showed a significant 1.3-fold increase (6.68 vs 5.12) in their mean ROY in comparison to female (*P* = 0.0014, Mann-Whitney test) (*SI Appendix,* Fig. S2C). However, when analyzed by academic ranks we found no significant differences in the scholarly output (ROY) nor in the *C* score of younger generations (Fig. 4D). These results are consistent with the notion that increased collaborative efforts across national and international borders can neutralize gender gaps in research productivity.

**Figure 5.**
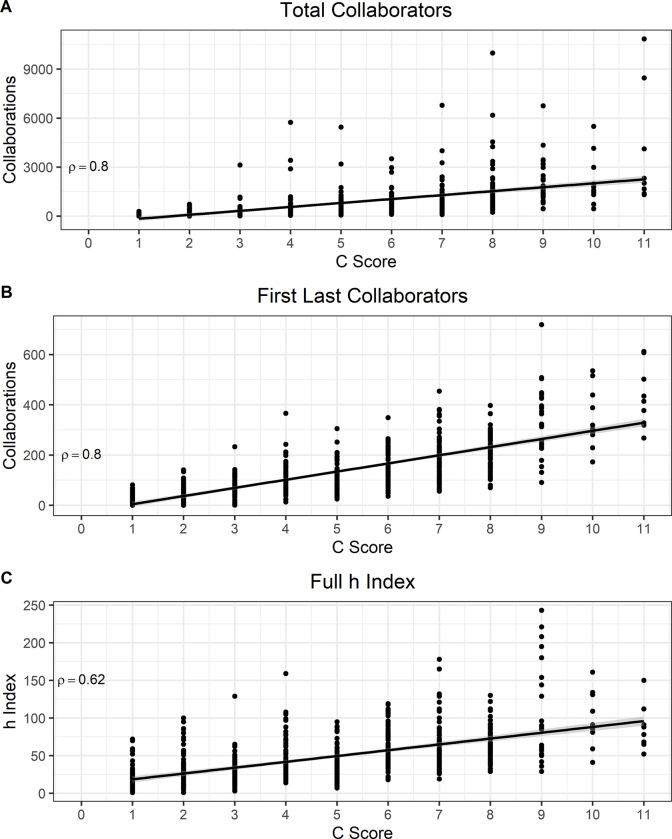
The *C* score demonstrates nurture of large and sustained collaborative networks. **A)** Total number of unique collaborators indicates a strong correlation with the *C* score. **B)** Number of unique First and Last author collaborators, indicative of efficient collaborative tendencies, reveal a very strong correlation with the *C* score. **C)** The *h* index and *C* score of all individuals included in this study show a strong and positive correlation.

Lastly, we determined whether the *C* score correlates with unique names of collaborators with the prediction being that investigators with higher *C* value would have more unique names identified with them. As expected, we found a very strong and positive correlation between the total number of unique collaborators and the *C* score, (ρ = 0.8, *N* = 579, *P* < 0.0001) (Fig. 5A). Given that the more publications people have the more likely they are to have unique collaborators associated with them, this strong association may not be indicative of large and sustained collaborative networks. Therefore, we limited the analysis to first and last authors considering that collaborative efforts between the lead and senior authors of a publication are more likely to suggest direct work between these individuals. This analysis indeed revealed a very strong correlation between the *C* score and the total number of unique first and last authors within a scientist’s publications (ρ = 0.8, *N* = 579, *P* < 0.0001) (Fig. 5B). In agreement with our initial hypothesis, the relationship between the *C* score and *h* index score of all investigators included in this study demonstrated a strong, positive correlation between the two variables (ρ = 0.62, *N* = 579, *P* < 0.0001) (Fig. 5C). We considered the possibility that the association between the *C* score and the *h* index was simply a function of the ROY variable dependence. To validate or refute this possibility we recalculated the *C* score based only the CN data (Reduced *C* score, *RC* score) and found that the correlation between the *h* index and *RC* score still demonstrated a strong to very strong relationship between the CN variable and *h* index disproving the *C* score hypothetical dependency of ROY (*SI Appendix,* Fig. S3–S6). These findings imply that the *C* score provides a simple parameter to assess academic performance acknowledging both scholarly output (ROY) and collaborative efforts (CN).

## Discussion

Competition is a self-limiting process in nature mostly driven by a shortage of resources (20). The unhealthy and hypercompetitive culture within the scientific enterprise rewarded by its winner-takes all economic system further reinforces the priority rule and in the long run can undermine public confidence in the scientific method (10, 21). To prevent signs of dysfunction that may jeopardize the integrity of science itself, it is critical to promote and reward collaborative efforts seeking to address perplexing questions and solve complex problems. As previously argued by Casadevall and Fang (22), perhaps an initial step towards recognizing science as an interconnected and cooperative community is at the level of academic promotions and scientific award committees reviewing an individual’s “scientific civility” as well as scientific performance.

We propose the *C* score as an index that measures an individual’s ability to nurture, foster, and develop extensive, sustained, and multicultural collaborations over time, leveraged on the strengths and expertise of professionals trained in different fields. We justify a 15-year timeframe (split into 12-year and a 3-year periods) to assess the collaborative networks of an individual based on an average of 5 years to achieve a milestone within an academic career: from receiving a Ph.D. to becoming an established Professor at an academic institution. During the length of an academic career, most academic scientists are immersed in teaching and learning with recurring roles as mentee and mentor. In this study, we focused on full-time faculty members with a tenure status (i.e. Assistant Professor, Associate Professor, and Professor) in an academic institution with very high research activity (23). The 12-year timeframe attempts to assess collaborative efforts with other scientists who may have been Ph.D. and/or post-doctoral supervisors, post-doctoral peers, mentees or contemporary colleagues at early tenure stages among others; while the 3-year timeframe endeavors to measure the development of collaborative projects with current Ph.D. and post-doctoral mentees, previous Ph.D. and post-doctoral mentees who have become independent researchers, and individuals from different areas of specialization with new perspectives to tackle an old problem or solve a new one.

In the workplace, productivity refers to how much work (output) is generated over a specific time. In academia, the traditional gold-standard to measure productivity of an academic scientist is the number of publications (Research Output) generated during the length of their career, which contributes to increased knowledge. One can make a meaningful contribution to the body of knowledge by using different approaches (points of view) to solve an identified problem. An effective and highly successful way to do this is by catalyzing collaborative efforts that bring together researchers with diverse scientific backgrounds and perspectives. Each of these individuals correspond to a network node capable of receiving, transmitting, or creating new knowledge itself. Networks also provide opportunities for greater efficiency since collaborating investigators contribute to areas where they have a particular expertise or technology. As we study here, growth and expansion of these networks is a function of time based on a scientist’s values and personal ambitions.

Some caveats in our analysis were noted. Our *C* score calculation between the academic ranks analyzed in this study was not adjusted to the appointment year since that information is not publicly available. We anticipate that accounting for it would provide a clearer picture of an individual’s current networks activity. For the estimation of academic career length, we used the very first paper published, which may not reflect the appropriate starting point if it represents a small contribution in a field with which one is no longer involved. The *C* score distribution in social sciences such as Economics tends to the lower end mainly attributed to a low ROY in the form of publications. The *C* score also cannot predict whether collaborative networks are forged in response to selfish and malicious competition or cooperative and joyful interactions between investigators. We caution readers about drawing interpretations between the gender differences noted here since their significance, if any, requires further study. For example, mindful of the limitations of assigning binary genders, an analysis of first author sharing patterns revealed a preference for male associations in sharing credit (24). Likewise, women in research teams are less likely than men to be credited with authorship which could translate in an apparently higher male productivity and thus affect *C* score calculations (25). Finally, the importance or significance of the publications (i.e., number of citations) used in calculating the *C* score is not considered in the calculation. Our metric did not computed journal impact factors.

Despite the foregoing limitations, unlike the *h* index that captures a passive cumulative impact of an author’s scholarly performance by considering publications and citations during the whole length of their academic life (14), the *C* score exhibits a dynamic behavior. It can decrease, increase, or remain unchanged over time since it responds to levels of engagement with collaborative projects, which reflects the cumulative relevance and productivity of an individual at a defined point of their academic career. Therefore, a high *C* score can be assumed as an indicator of an accomplished and collaborative scientist, but the opposite is not necessarily correct. A yearly calculated and averaged *C* score can provide a better indicator of consistencies in “scientific civility” over the lifetime of a scientist. Within an academic environment, the *C* score could provide a valuable tool to assess the upward trajectory of a scientist’s career from Assistant Professor to Professor. For example, if the *C* score of an individual is lower than expected for their cohort during professorial rank progression that could alert one to the need for an intervention to ensure continued academic success in the form of additional mentoring and advice.

The *C* score may reflect the ability of scientists to build and sustain dynamic working groups based on trust, consistency, and clear communication. Individuals with higher *C* scores are perhaps more likely to think about team science (12), and possess strong communication skills that promote competition while containing conflict to bolster the emergence of creative and multicultural cooperative solutions. These components of scientific productivity, generativity, and leadership require a strong foundation of civility. The *C* score gauges a scientist from an integral perspective as an individual who can generate knowledge in a cooperative and sustained manner, explaining the additive and synergistic interaction between the SOY and CN variables. This indicator could potentially provide a single-number criteria to assess academic performance in terms of a scientist’s active collaborative networks that prevail over institutionally limited economic resources and avoids inequalities related to the length of an individual’s academic career, field of investigation, and gender. The *C* score parameter also reflects the likelihood that an individual will be highly recognized by their scientific achievements and that comparisons in productivity between men and women can be avoided. Unlike other metric systems that assess a researcher’s scientific impact estimating the specific contribution (relative credit share) among coauthors (26), the *C* score demonstrates a simple approach to determine engagement and efficient collaborative tendencies by analyzing a scientist’s collaborative behavior.

In conclusion, we propose the *C* score as a new research indicator for evaluating the academic performance of an individual in terms of scholarly output and collaborative efforts at a certain timepoint of their career. This parameter can be adapted and adjusted to the most significant output of an academic institution whether it is research-, teaching-, or practice-related. For administrators and other professionals in decision-making scenarios, the *C* score, along with other indicators such as the *h* index, would support a more integrative and holistic assessment of an individual’s academic performance.

## Supplementary Material

Supplement 1 Figure S1. The *C* score is a potential tool for assessing career advancement in the academic environment from Assistant Professor to Professor.
**Figure S2. Metrics to assess research productivity exhibit a gender gap. A)** *h* index, **B)** *C* score, and **C)** Research Output normalized by total number of career years.
**Figure S3. The Reduced *C* score could be a useful index to assess academic performance.** A scientist’s h index shows a positive strong relationship with the Reduced C score. **A)** Case study of The Johns Hopkins Bloomberg School of Public Health (BSPH). **B)** Case of 10 individual departments that comprise the BSPH. **C)** Profile by grouping departments according to the type of laboratory environment. **D)** The Reduced *C* score demonstrates a weak relationship with the length of an individual’s academic career. **E)** The mean Reduced *C* score increases as the academic career of an individual advances from Assistant Professor to Professor.
**Figure S4. The Reduced *C* score reflects the likelihood of being recognized by a prestigious award and does not reflect a cumulative pattern. A)** Case study of Nobel Laureates from 2010-2020 in four categories. **B)** Individual categories of Nobel Prizes also demonstrated a strong association between the two variables.
**Figure S5. Active collaborative networks can bolster an individual’s academic performance. A)** Case study of ASM International Fellows from developing countries. **B)** Case study of retracted authors. **C)** Academic performance in terms of the Reduced *C* score demonstrates no gender inequalities. **D)** Gender inequalities disappear across all professorial ranks when academic performance in exclusively assessed in terms of collaborative efforts.
**Figure S6. The Reduced C score demonstrates nurture of large and sustained collaborative networks. A)** Total number of unique collaborators indicates a very strong correlation with the Reduced *C* score. **B)** Number of unique First and Last author collaborators, indicative of efficient collaborative tendencies, reveal a very strong correlation with the Reduced *C* score. **C)** The *h* index and Reduced *C* score of all individuals included in this study show a strong and positive correlation.
**Table S1.** Nobel Laureates included in this study. The C score was calculated for 103 awardees from Chemistry, Physics, Economics, and Physiology categories during 2010 to 2020. DOI 10.6084/m9.figshare.21957110.
**Table S2.** Master database used for this study. DOI 10.6084/m9.figshare.21957014

## Significance Statement

The increased connectivity between fields and specialties of science is undeniable. We propose a new indicator, the *C* score, to assess the value of collaborative efforts and research output to a scientist’s academic performance. This indicator reflects collaborative and interdisciplinary efforts and provides a measure of “scientific civility” and teamwork. The *C* score can be used as a decision-making tool to track career advancement within the academic environment. Along with other indicators such as the *h* index, the *C* score supports a more integrative and holistic assessment of an individual’s academic performance.
