# Supplementary figures and images for: Scientific civility and academic performance
Source: bioRxiv. 2024 Jan 5:2023.01.26.525747. Originally published 2023 Jan 27. Preprint. [Version 2] doi: 10.1101/2023.01.26.525747 (PMC9900961; doi:10.1101/2023.01.26.525747)

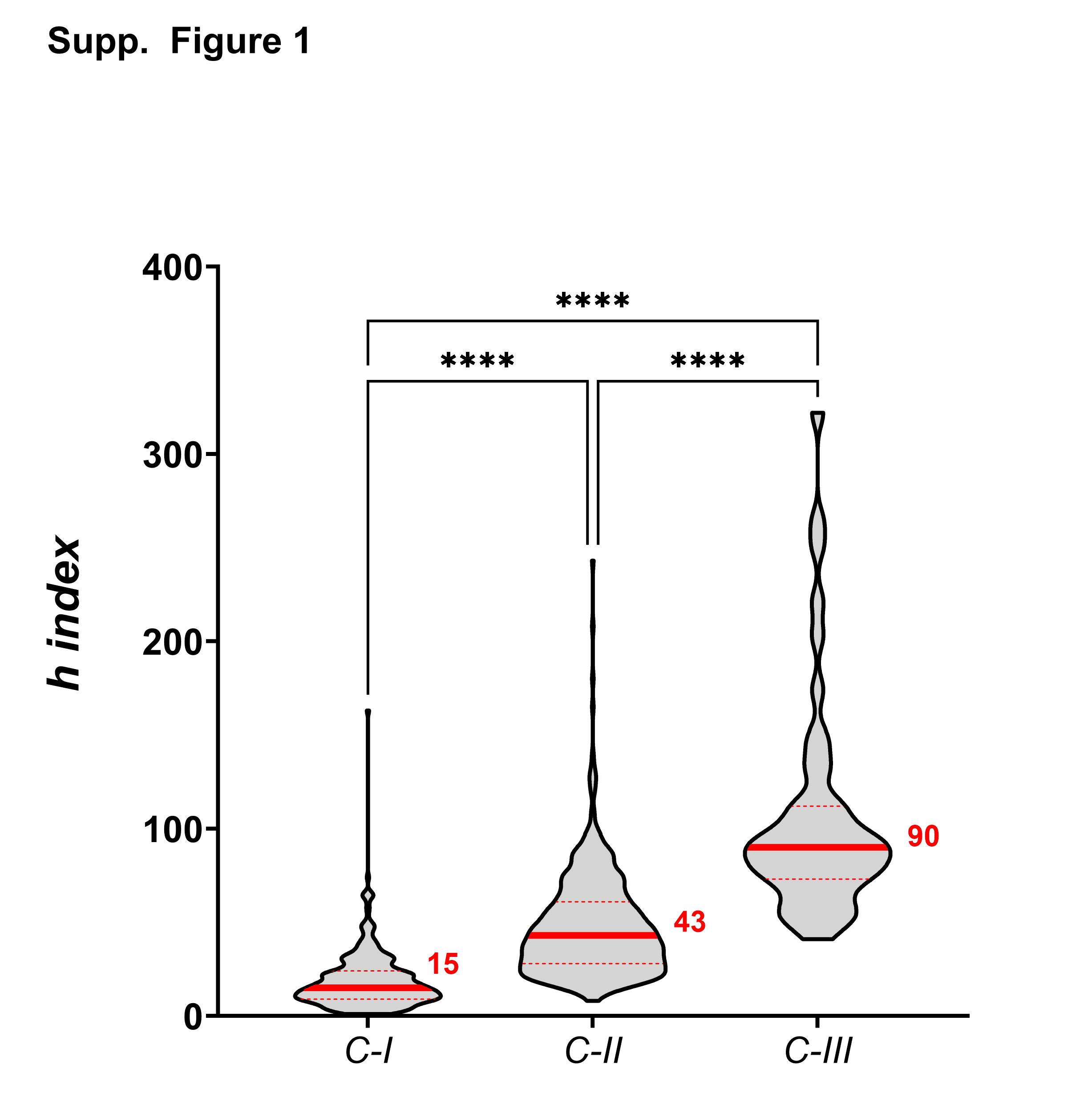

Supplement: Supplement 1 [file media-1.jpg]
