# Supplementary material for: Scientific civility and academic performance
Source: bioRxiv. 2023 Jan 27:2023.01.26.525747. Preprint. [Version 1] doi: 10.1101/2023.01.26.525747 (PMC9900961; doi:10.1101/2023.01.26.525747)
Supplement: Supplementary file 1 [file media-1.pdf]

Figure S1

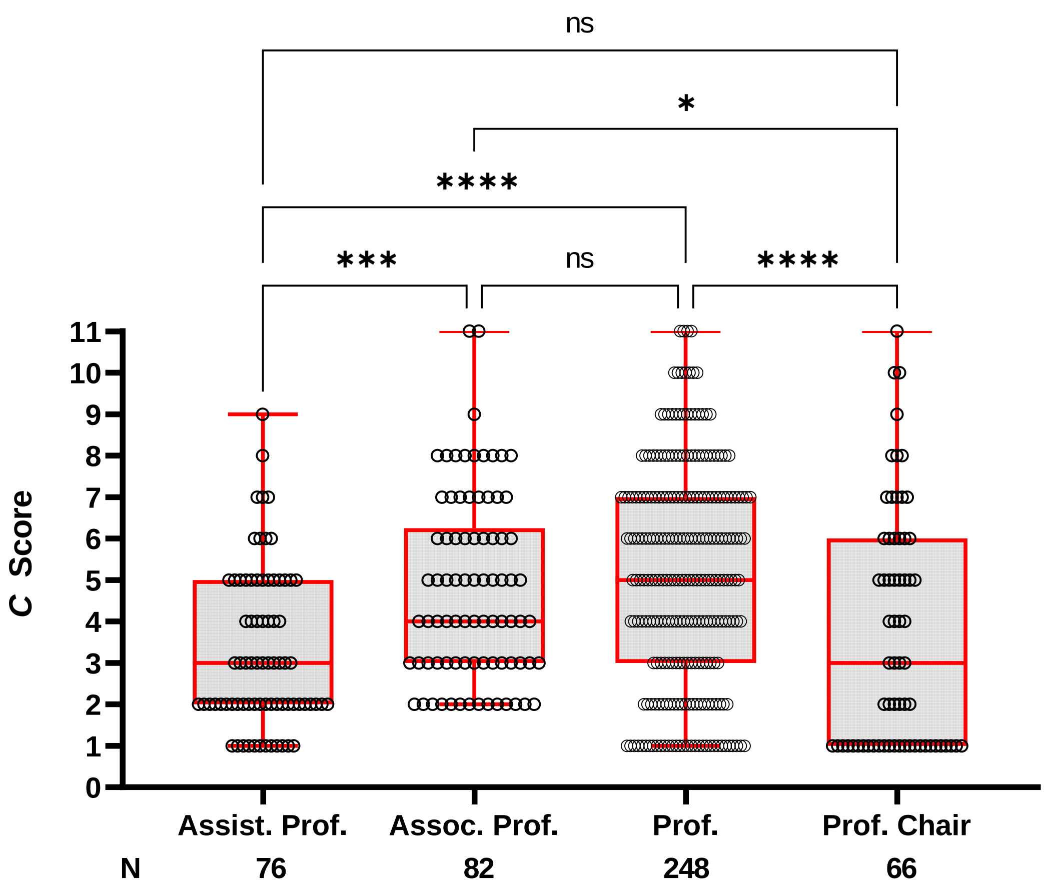

ns
*
****
***
ns
****
C Score
0
1
2
3
4
5
6
7
8
9
10
11
Assist. Prof.
Assoc. Prof.
Prof.
Prof. Chair
N
76
82
248
66

Figure S2

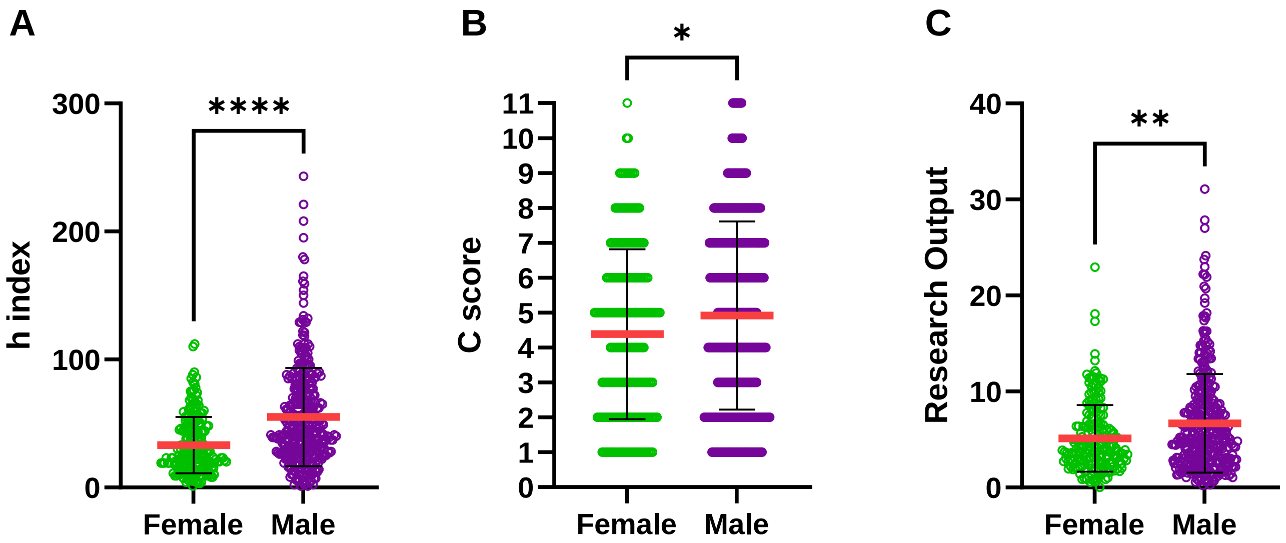
A
h index
300
200
100
0
****
Female
Male
B
C score
11
10
9
8
7
6
5
4
3
2
1
0
*
Female
Male
C
Research Output
40
30
20
10
0
**
Female
Male

Figure S3

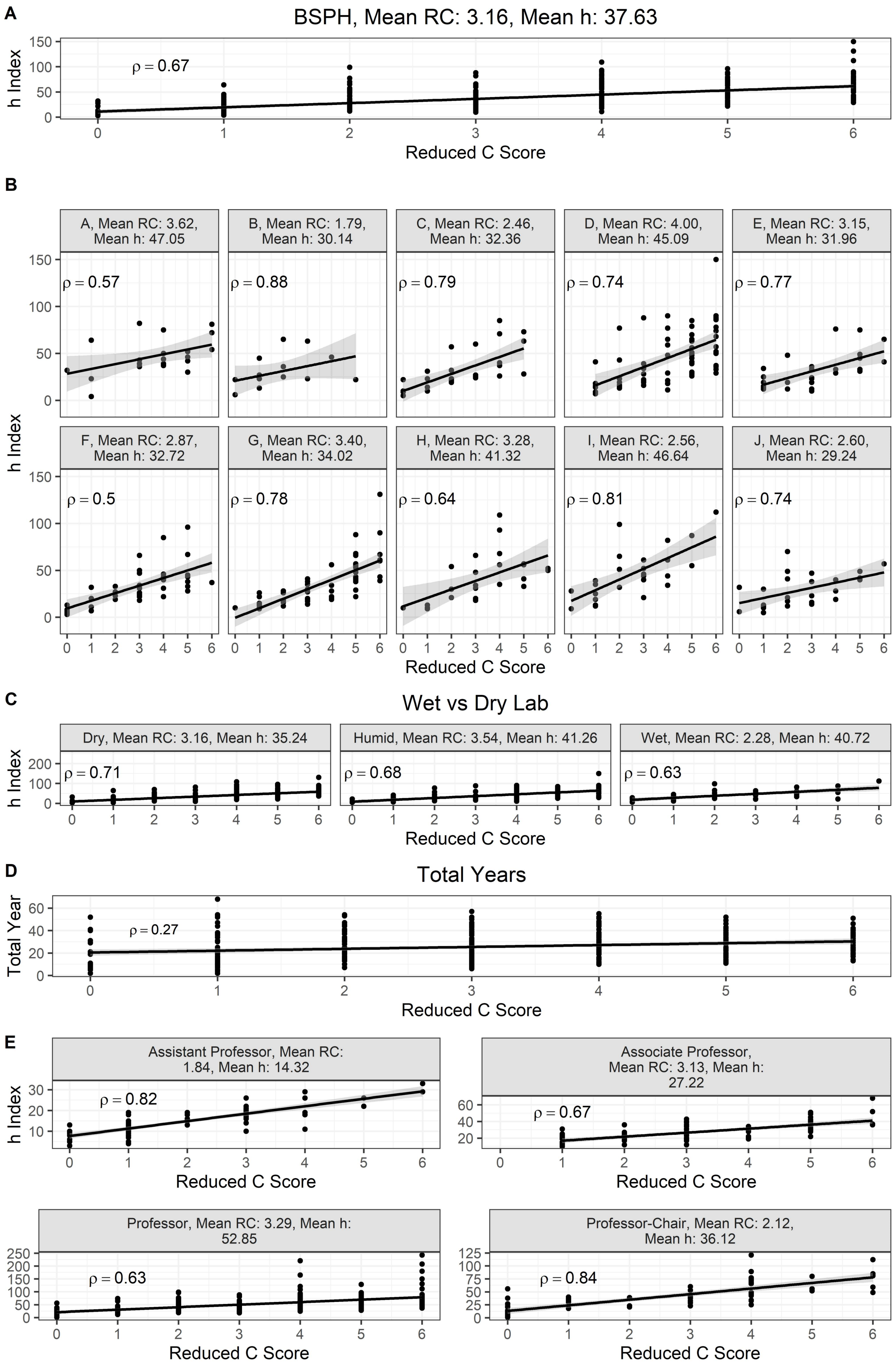
A
BSPH, Mean RC: 3.16, Mean h: 37.63
ρ = 0.67
h Index
Reduced C Score
B
A, Mean RC: 3.62, Mean h: 47.05
ρ = 0.57
B, Mean RC: 1.79, Mean h: 30.14
ρ = 0.88
C, Mean RC: 2.46, Mean h: 32.36
ρ = 0.79
D, Mean RC: 4.00, Mean h: 45.09
ρ = 0.74
E, Mean RC: 3.15, Mean h: 31.96
ρ = 0.77
F, Mean RC: 2.87, Mean h: 32.72
ρ = 0.5
G, Mean RC: 3.40, Mean h: 34.02
ρ = 0.78
H, Mean RC: 3.28, Mean h: 41.32
ρ = 0.64
I, Mean RC: 2.56, Mean h: 46.64
ρ = 0.81
J, Mean RC: 2.60, Mean h: 29.24
ρ = 0.74
h Index
Reduced C Score
C
Wet vs Dry Lab
Dry, Mean RC: 3.16, Mean h: 35.24
ρ = 0.71
Humid, Mean RC: 3.54, Mean h: 41.26
ρ = 0.68
Wet, Mean RC: 2.28, Mean h: 40.72
ρ = 0.63
h Index
Reduced C Score
D
Total Years
ρ = 0.27
Total Year
Reduced C Score
E
Assistant Professor, Mean RC: 1.84, Mean h: 14.32
ρ = 0.82
Associate Professor, Mean RC: 3.13, Mean h: 27.22
ρ = 0.67
Professor, Mean RC: 3.29, Mean h: 52.85
ρ = 0.63
Professor-Chair, Mean RC: 2.12, Mean h: 36.12
ρ = 0.84
h Index
Reduced C Score

Figure S4

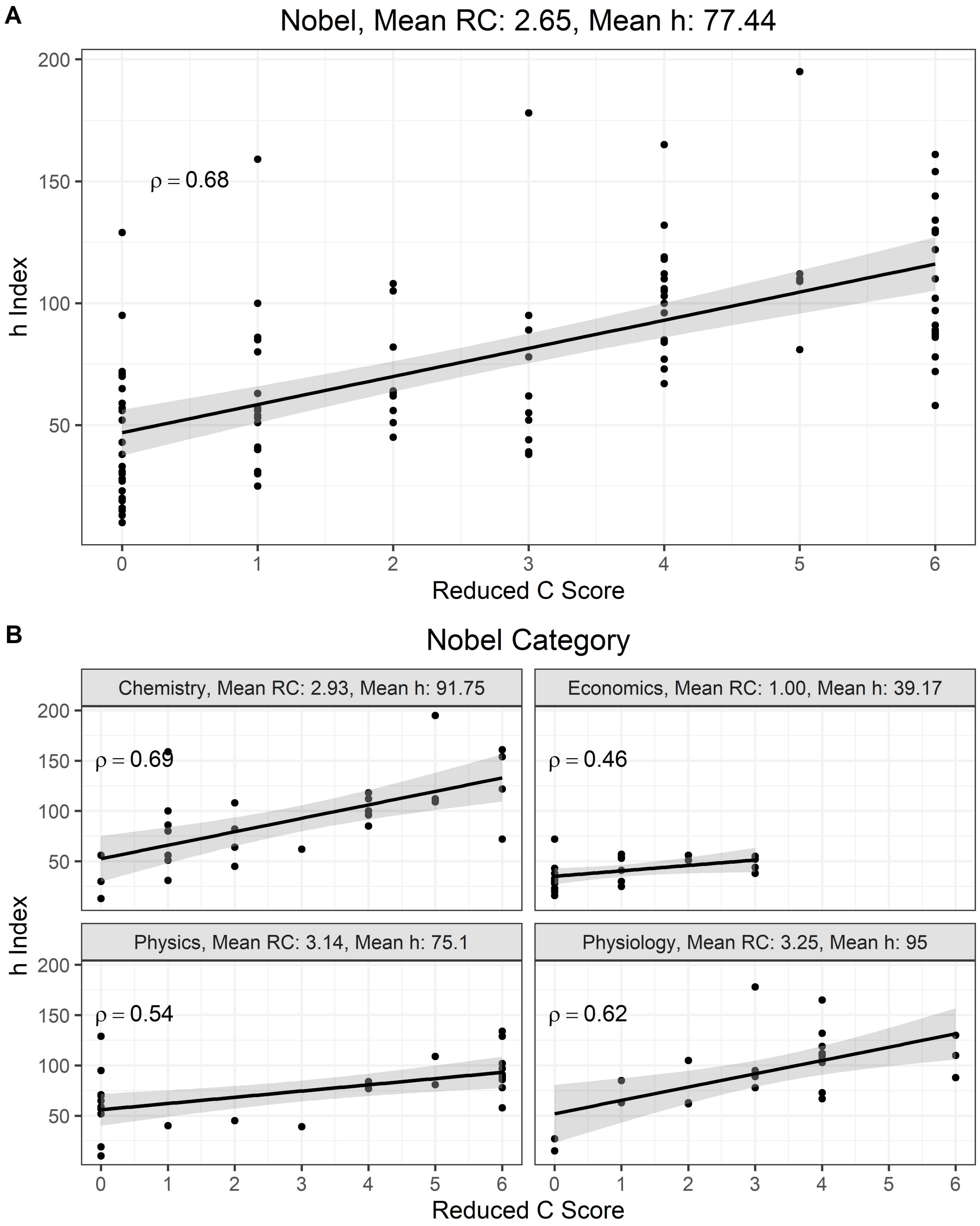
A
Nobel, Mean RC: 2.65, Mean h: 77.44
ρ = 0.68
h Index
Reduced C Score
B
Nobel Category
Chemistry, Mean RC: 2.93, Mean h: 91.75
ρ = 0.69
Economics, Mean RC: 1.00, Mean h: 39.17
ρ = 0.46
Physics, Mean RC: 3.14, Mean h: 75.1
ρ = 0.54
Physiology, Mean RC: 3.25, Mean h: 95
ρ = 0.62
h Index
Reduced C Score

Figure S5

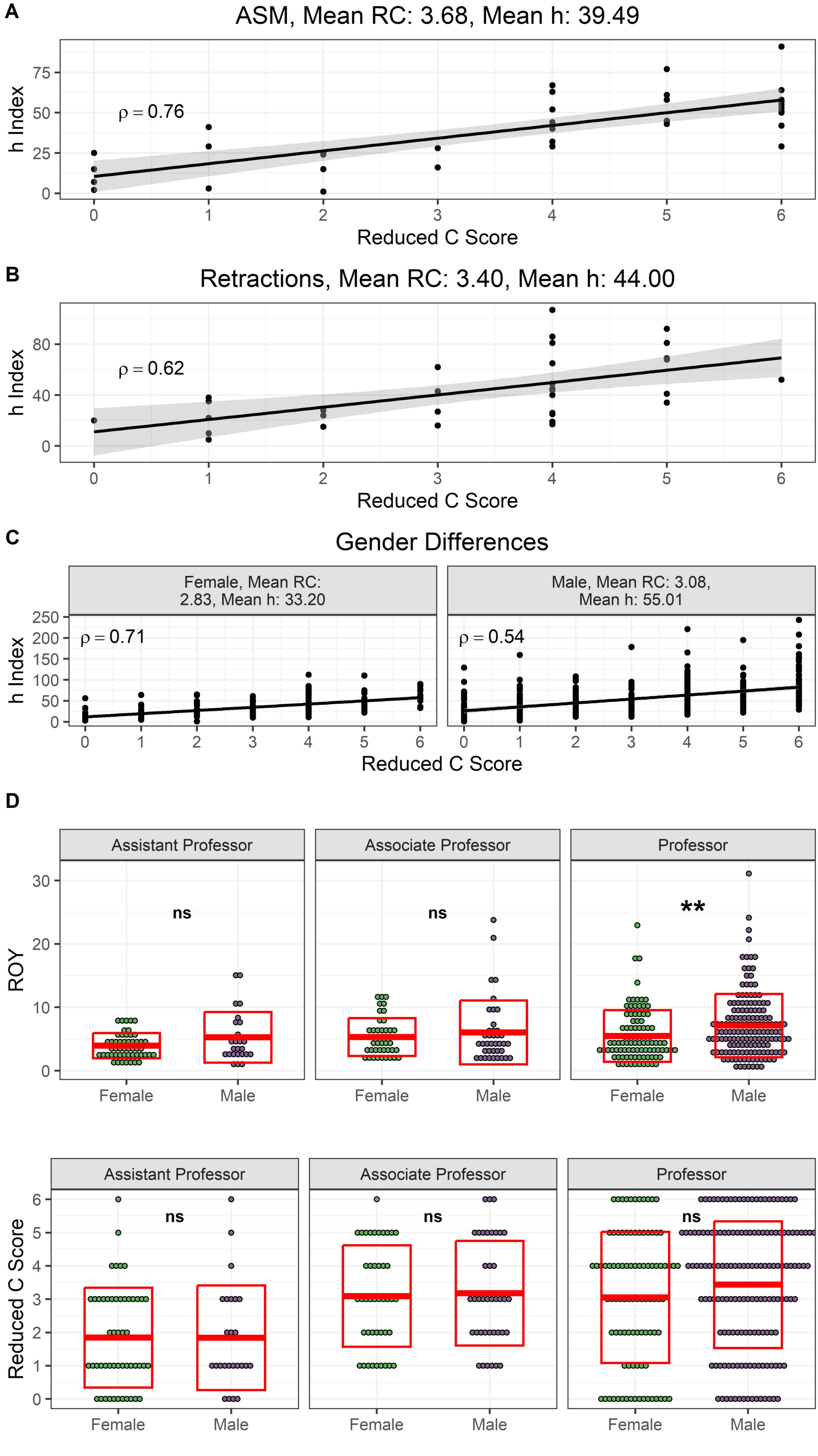

A
ASM, Mean RC: 3.68, Mean h: 39.49
h Index
ρ = 0.76
Reduced C Score
B
Retractions, Mean RC: 3.40, Mean h: 44.00
h Index
ρ = 0.62
Reduced C Score
C
Gender Differences
Female, Mean RC: 2.83, Mean h: 33.20
Male, Mean RC: 3.08, Mean h: 55.01
h Index
ρ = 0.71
ρ = 0.54
Reduced C Score
D
Assistant Professor
Associate Professor
Professor
ROY
ns
ns
**
Female
Male
Reduced C Score
ns

Figure S6

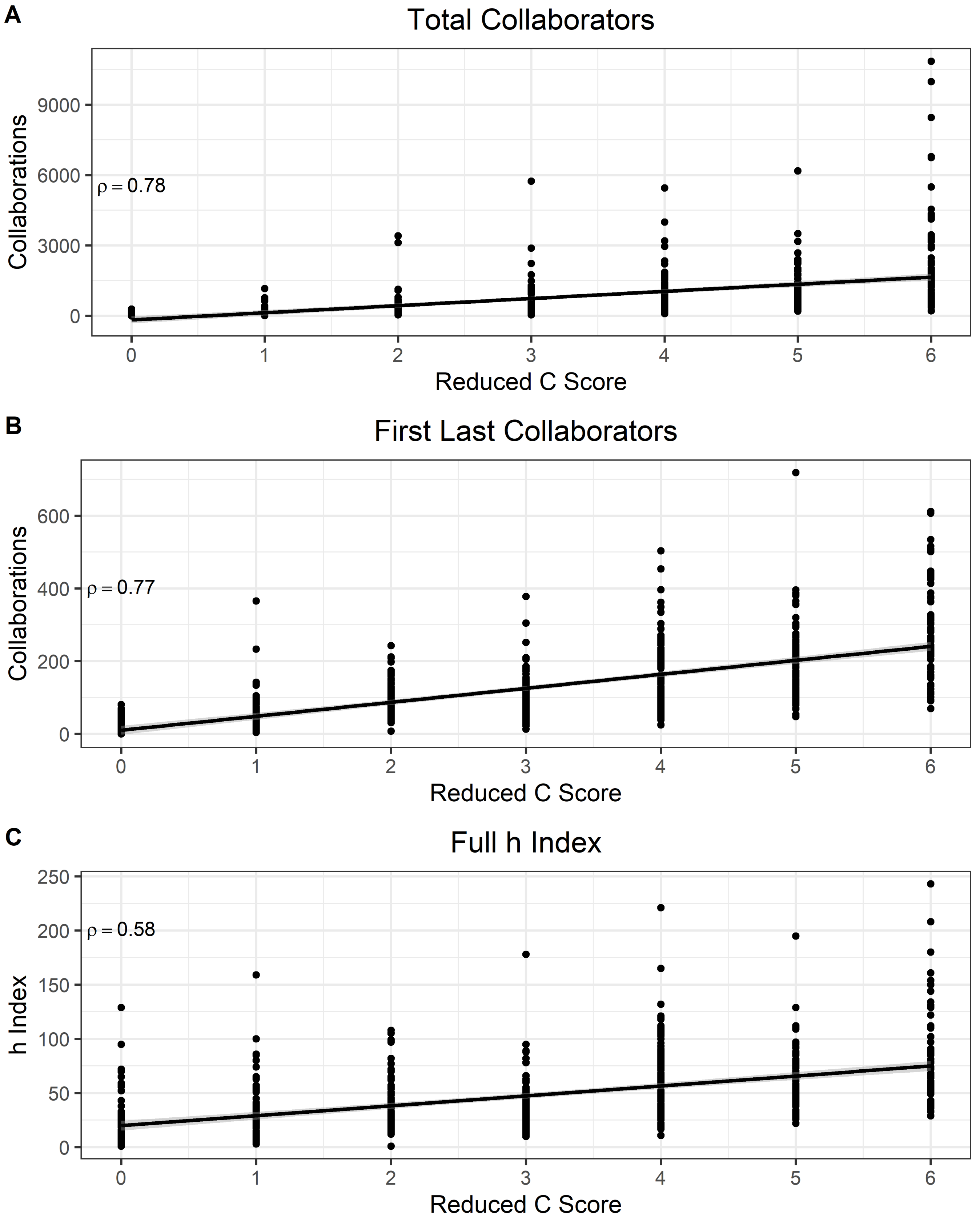
A
Total Collaborators
Collaborations
ρ = 0.78
9000
6000
3000
0
0
1
2
3
4
5
6
Reduced C Score
B
First Last Collaborators
Collaborations
ρ = 0.77
600
400
200
0
0
1
2
3
4
5
6
Reduced C Score
C
Full h Index
h Index
ρ = 0.58
250
200
150
100
50
0
0
1
2
3
4
5
6
Reduced C Score
